# Analysis of a Smart Sensor Based Solution for Smart Grids Real-Time Dynamic Thermal Line Rating

**DOI:** 10.3390/s21217388

**Published:** 2021-11-06

**Authors:** Yuming Liu, Jordi-Roger Riba, Manuel Moreno-Eguilaz, Josep Sanllehí

**Affiliations:** 1 Campus Terrassa, Universitat Politècnica de Catalunya, Rambla Sant Nebridi 22, 08222 Terrassa, Spain; yuming.liu@upc.edu (Y.L.); manuel.moreno.eguilaz@upc.edu (M.M.-E.); 2 SBI Connectors, Albert Einstein 5, 08635 Sant Esteve Sesrovires, Spain; josep.sanllehi@sbiconnect.es

**Keywords:** wind speed, dynamic thermal line rating, ACSR conductor, real-time monitoring, wireless communications

## Abstract

Dynamic thermal line rating (DTLR) allows us to take advantage of the maximum transmission capacity of power lines, which is an imperious need for future smart grids. This paper proposes a real-time method to determine the DTLR rating of aluminum conductor steel-reinforced (ACSR) conductors. The proposed approach requires a thermal model of the line to determine the real-time values of the solar radiation and the ambient temperature, which can be obtained from weather stations placed near the analyzed conductors as well as the temperature and the current of the conductor, which can be measured directly with a *Smartconductor* and can be transmitted wirelessly to a nearby gateway. Real-time weather and overhead line data monitoring and the calculation of DTLR ratings based on models of the power line is a practical smart grid application. Since it is known that the wind speed exhibits important fluctuations, even in nearby areas, and since it plays a key role in determining the DTLR, it is essential to accurately estimate this parameter at the conductor’s location. This paper presents a method to estimate the wind speed and the DTLR rating of the analyzed conductor. Experimental tests have been conducted to validate the accuracy of the proposed approach using ACSR conductors.

## 1. Introduction

With the widespread deployment of heat pumps, electric vehicles, and different electric and electronic technologies, the consumption of electrical power is increasing steadily, so there is a need to increase the capacity of existing power lines. However, any increase of the transmission capacity must not compromise safe operation, supply security, and reliability [1].

High-voltage overhead transmission lines typically use aluminum conductor steel-reinforced (ACSR) cables [2]. It is known that due to the steel core, ACSR conductors have a larger ac/dc resistance ratio compared to all-aluminum conductors due to the magnetic induction in the steel core. This magnetic induction causes power losses due to the induced eddy currents and the hysteresis effect and redistributes the current in the aluminum wires layers [3].

The allowable conductor temperature limits the load or current capacity of the power line, so the operating temperature must be restricted to below the allowable operating temperature to limit the ground clearance of the conductors [4]. Dynamic thermal line rating (DTLR) offers a solution to this problem because it is a smart and cost-effective solution for utilizing the maximum ampacity or ampere capacity of transmission lines [5], which differs from static line rating (SLR), the conventional and simple approach, which is based on conservative criteria [5] that represent severe or worst case weather conditions [6]. SLR calculates the ampacity of the line from deterministic or probabilistic methods to determine the atmospheric operating conditions, which have a heavy influence. SLR often results in a conservative rating because it determines the same ampacity limit for the whole year; it is a static value, regardless of current weather conditions. Conversely, DTLR is based on measuring the weather variables, so the maximum allowable current of the line is dynamically calculated to ensure that the line operates within safe operation limits. Therefore, DTLR requires the current and temperature of the line and the weather variables in the vicinity of the power line to be monitored online using specific sensors and weather stations [1]. By applying a DTLR approach, the maximum rating or ampacity can be calculated from the mathematical line models that can be found in [7,8], with the results being greatly influenced by the current weather conditions. The current carrying capacity or ampacity of overhead power conductors can be affected by many factors such as wind speed, wind direction, solar radiation, and ambient temperature. Among these factors, wind speed is significant in terms of ampacity calculation [9,10].

Different DTLR approaches can be found in the technical literature. According to [9], DTLR methods can be roughly classified into indirect and direct methods. Indirect methods estimate the thermal line rating from the weather data gathered from weather stations or that have been forecasted, representing the main inputs of the method. These methods determine the required thermal rating based on solving the conductor heat balance equation, as detailed in Cigré [7], IEEE [8], or IEC [11]. Direct methods for dynamic line rating directly measure physical power line variables, including conductor temperature or/and current, line mechanical tension, conductor sag, or ground clearance, as described in [6]. Since there is no need to install weather measuring devices on the line and since they are reliable and not very expensive, indirect methods are simpler and present lower costs compared to direct methods, so indirect methods are indicated for power lines that are relatively light load. Compared to direct methods, indirect methods are less accurate because the conductor temperature and line ampacity are estimated indirectly using theoretical models [12]. Conversely, direct methods rely on field data; thus, they can be more accurate since no relationship between conductor temperature and the measured data from indirect methods is needed [9].

The fast progress made in the development of communication systems, sensors, and control algorithms has led to the development of smart grids, which integrate distributed energy resources, loads, energy storage, and control systems. They present substantial advantages, such as enhanced power supply reliability, reduced power losses, energy independence, and the integration of renewable energy sources [13]. To this end, smart grids integrate information technology to share power data in real-time for the efficient management of the power demand to maximize power efficiency, so DTLR methods represent a key element for smart grid development [14,15,16,17,18,19,20]. Recent studies have suggested that IoT solutions allow smart grid reliability to be enhanced while also remarkably improving their capacity of [21,22,23,24].

Nowadays, DTLR is a hot topic because of the widespread use of accurate, reduced-size, and cost-effective sensors; the development of several communication systems that are compatible with high-voltage applications; the need to expand power transmission capability; and the fact that DTLR allows the ampacity of overhead power lines to be improved through the measurement of the line and weather variables.

In [25], wind speed and DTLR ampacity are estimated by measuring different parameters such as the conductor current, temperature, and mechanical tension; ambient temperature; and solar radiation and by applying the sag-tension method. Sag-tension monitoring methods require precise state change equations to relate the conductor temperature to the sag-tension [26]. In [27], the DTLR rating of a distribution line was calculated using a low cost sensing probe to measure the conductor temperature and to transmit the data wirelessly. However, the line current was not measured in real-time, which is an important parameter in this application [26]. In [28], a self-powered high-voltage sensor is presented that measures line temperature, voltage, current, and the active and reactive power to determine the SLR and DTLR ratings. It also requires environmental data such as average wind speed and direction or air pressure from local weather stations. Nevertheless, the average wind speed taken from nearby weather stations is often not accurate, as wind speed changes with terrain topography and vegetation. In [29], a reverse calculation is presented to estimate the wind speed from an online conductor current and temperature, solar radiation, and ambient temperature measurements, but the paper does not present estimates of the DTLR rating.

This paper presents an approach to estimate the DTLR rating of power lines based on ACSR conductors, combining the real-time monitoring of weather and line data. It is a practical smart grid application since the proposed DTLR approach allows the power lines to operate at their maximum capacity by adapting the rating according to the current real-time weather conditions. Solar radiation and ambient temperature are important variables that can be used to determine the maximum allowable power transmission conductor current. Nevertheless, in this paper, they are not directly measured. Instead, such variables are obtained from a nearby weather station. The principal reason for this is because of the similarities between the ambient temperature and the solar radiation measured by the weather station and the local values at the conductor’s surface. Secondly, there is a need to simplify the system with the purpose of reducing the power consumption and the cost of the sensors installed in the high-voltage conductors. Finally, wind speed plays a much more significant role than that of ambient temperature and solar radiation in terms of DTLR calculation [9]. The proposed method presents several novelties and contributions. First, it develops the *Smartconductor* prototype, which measures the current and temperature of the conductor in real-time. Second, it requires reduced computational resources and presents a low computational burden to minimize the hardware requirements for compatibility, only requiring inexpensive devices global smart grid deployment. Third, the proposed method estimates the wind speed; thus, there is no need to use a wind speed sensor. Since the DTLR rating depends heavily on the local wind speed and since the wind speed has an important cooling effect, it is estimated based on a reverse calculation by applying a thermal model of the ACSR conductor. Once the wind speed has been estimated, the DTLR rating is calculated from the thermal model. Fourth, the proposed method estimates the joule and magnetic losses of the ACSR conductor from the measured ac resistance of the conductor, this being another contribution of the paper. The proposed approach has been validated under different operating conditions by means of experimental tests by considering different controlled wind speeds.

The experimental results prove that the real-time approach presented in this paper can predict both the value of the local wind speed and the DTLR rating with accuracy and with a reduced computational burden, so the calculations can be implemented in the low-power microprocessors that are used in inexpensive devices that are required for global smart grid deployment. Therefore, the developments made in this paper contribute the research area focusing on smart grids. The proposed DTLR approach allows us to take advantage of the maximum transmission capacity of power lines by adapting the rating of the line according to the current weather conditions in real-time, making it a smart solution of paramount importance in future smart grids.

Section 2 describes the *Smartconductor* device, including its sensors and wireless communications. Section 3 details the equations required to estimate the wind speed and the dynamic thermal line rating. Section 4 outlines the strategy applied to estimate the wind speed and the dynamic thermal line rating and includes a flow chart detailing the full process. Section 5 describes the experimental setup, including the power source, conductors, sensors, and measuring devices. Section 6 presents and explains the results that were attained, and finally, Section 7 concludes the study.

## 2. *Smartconductor.* Sensors and Wireless Communications

This section describes the sensors used in the *Smartconductor* device as well as the wireless communications approach that is applied.

### 2.1. Current Sensor

Different sensor technologies can be applied to measure the current flowing through a conductor, such as giant magneto resistive, Rogowski coils, current transformers, or Hall effect sensors [30]. The Hall effect sensor was selected for the *Smartconductor* because this technology offers miniaturization, low power consumption, high linearity, and the possibility of sensing high magnetic fields. This sensor measures the magnetic flux density *B* that is generated by the conductor and generates an output voltage *V_Hall_* that is proportional to the measured magnetic flux density as described in (1):

(1) $$\;V_{Hall}=kB\;\left[V\right]\;$$

where *k* [V/T] is the sensitivity constant.

According to the Biot–Savart law [31], the magnetic flux density detected by a sensor placed on the top of a cylindrical conductor can be expressed as

(2) $$B=\frac{\mu_{0}I}{2\pi \left(r+h\right)}\;\left[T\right]\;$$

where μ_0_ = 4π10^−7^ H/m is the permeability of air, *I* (A) is the current in the conductor, *r* (m) is the radius of the conductor, and *h* (m) is the radial distance between the outer surface of the conductor and the sensor.

Hence, when placing the sensor on the surface of the conductor, the position *r + h* (m) is known as well as the magnetic flux density *B* in Equation (1), so the current *I* (A) through the conductor can be obtained as

(3) $$I=\frac{V_{Hall}\left(r+h\right)}{k\times 2\times 10^{-7}}\;\left[A\right]$$

### 2.2. Temperature Sensor

Since the conductor temperature is considered to be an essential parameter in determining the dynamic thermal line rating [32], it is of paramount importance to use a suitable temperature sensor. It should be considered that the maximum allowable temperature of the tested ACSR conductor for continuous operation is 90 °C [33]. Therefore, the temperature sensor should reach this range. When focusing on the expected linearity and accuracy and by taking the high current application into account, a positive temperature coefficient (PTC) resistor is a suitable choice, so a Pt1000 sensor was selected. When dealing with Pt1000 platinum sensors, each temperature value corresponds to exactly one resistance value, the correspondences can be tabulated in the EN 60,751 standard [34] as follows

(4) $$\;R_{T}=R_{0}\left(1+aT+bT^{2}\right)\;\mathrm{for}\;T\;>0\;{}^{\circ}C\;$$

(5) $$R_{T}=R_{0}\left(1+aT+bT^{2}+c\left(T-100\right)T^{3}\right)\;\mathrm{for}\;T<0\;{}^{\circ}C$$

where $\;a=3.9083\times 10^{-3}\;{}^{\circ}C^{-1},\;b=-4.183\times 10^{-7}\;{}^{\circ}C^{-1},c=-4.183\times 10^{-12}\;{}^{\circ}C^{-1},\;R_{0}=10^{3}\;Ω$, and $R_{T}$ is the resistance of the temperature sensor at the measured temperature in ohms.

### 2.3. Wireless Communications

The wireless communication of the proposed system is based on the Bluetooth SoC (System on Chip) nRF52832 from Nordic Semiconductors (Trondheim, Norway). This chip was selected since it contains an inbuilt BLE (Bluetooth Low Energy) module, inbuilt ADC converters, and low power consumption modes, and it is also inexpensive.

With respect to the gateway, after considering several features, such as cost and size, the Raspberry Pi 4 module was selected. It is worth noting that a Huawei e3372 LTE 4G Wi-Fi dongle was mounted in Raspberry Pi because 4G technology allows it to remotely control the Raspberry Pi and send data to the cloud.

**Figure 1:**
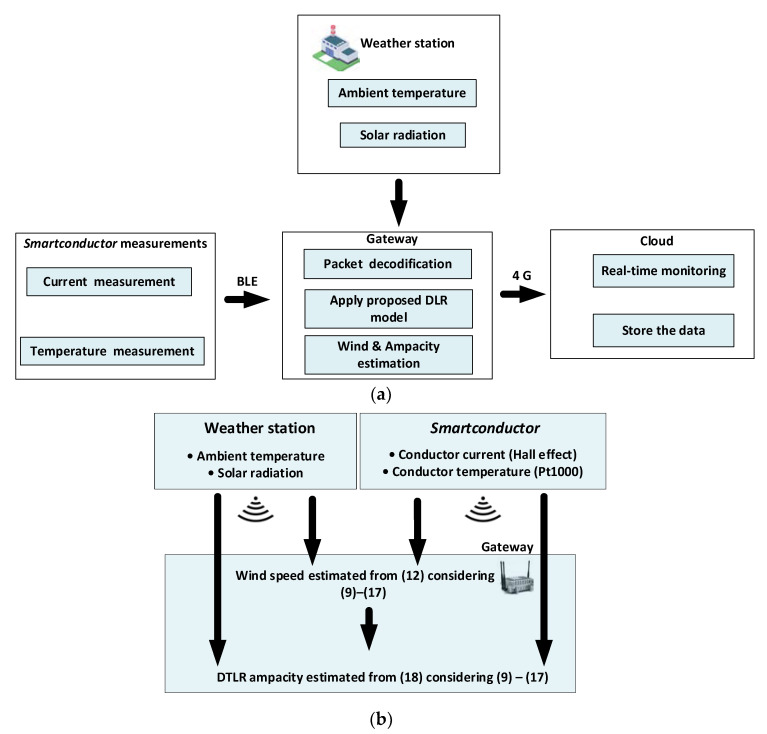
Proposed strategy to estimate the ampacity. (**a**) Global strategy. (**b**) Block diagram of the strategy to determine the DTLR rating.

Figure 1 shows the applied strategy to estimate the ampacity. To this end, the solar radiation and ambient temperature values are obtained from a nearby weather station, whereas the *Smartconductor* measures the conductor current and temperature. These values are sent wirelessly via BLE to the local gateway, which, in turn, sends the data to the cloud, where it is stored. The *Smartconductor* was programmed to connect to the gateway and to send the measured line current and conductor temperature values in a packet every 7 s via Bluetooth. Once the gateway receives the data by means of a python script implemented in the Raspberry Pi, the data that are received are decoded, and the ampacity is calculated. The proposed DTLR model takes the ambient temperature and solar radiation data from a nearby weather station, whereas the line current and the temperature of the conductor are directly measured by the *Smartconductor*. From these data, in the first stage, the wind speed is estimated, and in the second stage, the dynamic ampacity is estimated in real-time. Once the calculation is complete, the results are sent to a cloud server via 4G communication.

According to Figure 1b, in the first stage, the wind speed is determined from four readings (ambient temperature, solar radiation, conductor current and conductor temperature), and in the second stage, the DTLR rating is determined.

## 3. Dynamic Thermal Line Rating Estimation Method

The CIGRE standard [7] describes a non-steady-state equation using the following transient thermal balance equation based, which is expressed as

(6) $$P_{J}+P_{M}+P_{S}=P_{c}+P_{r}+mc\frac{dT_{c}}{dt}\;\left[W/m\right]\;$$

where $P_{J},P_{M},P_{S}$ are the heat gain terms due to joule, magnetic, and solar heating effects, respectively; $\;P_{c}\;\mathrm{and}\;P_{r}$ are the heat loss terms due to convection and radiation, respectively; *m* is the mass of the conductor in kg/m, *c* is the specific heat capacity of the conductor in J/(kg°C), and $T_{c}$ is the average conductor temperature in °C.

The heat capacity *c* of the ACSR conductor is calculated as follows:

(7) $$\{\begin{array}{c} mc=m_{Al}c_{Al}+m_{s}c_{steel}\\ c\left(T\right)=c_{20{}^{\circ}C}\left[1+\beta \left(T_{c}-20\right)\right] \end{array}\;$$

where *m_Al_* and *c_Al_* refer to the mass per unit length and the specific heat capacity of the aluminum part, respectively, whereas *m_steel_* and *c_steel_* refer to the mass per unit length and specific heat capacity of the steel part, respectively. The values of the temperature coefficient β are 3.8 × 10^−4^ °C^−1^ for pure Al, 4.5 × 10^−4^ °C^−1^ for the Al alloy and 1.0 × 10^−4^ °C^−1^ for steel [7].

According to [25], the joule and magnetic heat gains can be combined in only one equation, which appears as follows:

(8) $$P_{J}+P_{M}=I^{2}R_{ac}$$

where *I* is the root mean square (RMS) value of the current, amd $R_{ac}$ is the ac resistance of the conductor per unit length at the operating mean conductor temperature *T_c_*. The ac resistance of the conductor *R_ac_* includes the skin and proximity effects as well as the core losses, which can be calculated according to the method detailed in the Cigré Technical Brochure [35], or it can be measured. Measurements can be conducted according to the procedure described in [36] or in [31], with the last method being applied in this paper, a decision that is based on the previous experience of the authors.

As the ac resistance *R_ac_* is required during the process to determine the joule and magnetic heat gains, the conductor characteristic *R_ac_* (*T_c_*) was measured in the laboratory by measuring the temperature of the conductor, the voltage drop between two points of the conductor surface distanced by 1 m, and the ac current flowing through the conductor. Next by applying (9), the ac resistance was calculated as

(9) $$R_{ac}=\Delta V\cos\varphi /I\;$$

where φ is the phase shift between the voltage drop Δ*V* and the current *I* [31].

According to [7], Equations (10)–(15) are used to determine the heat loss due to convective cooling:

(10) $$P_{c}=\pi \lambda_{f}(T_{c}-T_{a})N_{u}\;\left[W/m\right]$$

where *λ_f_* = 2.42 × 10^−2^ + 7.2 × 10^−5^·*T_f_* in W/(m °C) is the thermal conductivity of air,$\;T_{c}$ is the conductor surface temperature, $T_{a}\;$is the ambient temperature, and *T_f_* is the film temperature defined as *T_f_* = 0.5(*T_a_* + *T_c_*).

Equation (9) applies for both natural and forced convective cooling, the difference between both situations is found in the way to allow the calculation of the de Nusselt number *N_u_*.

**Figure 2:**
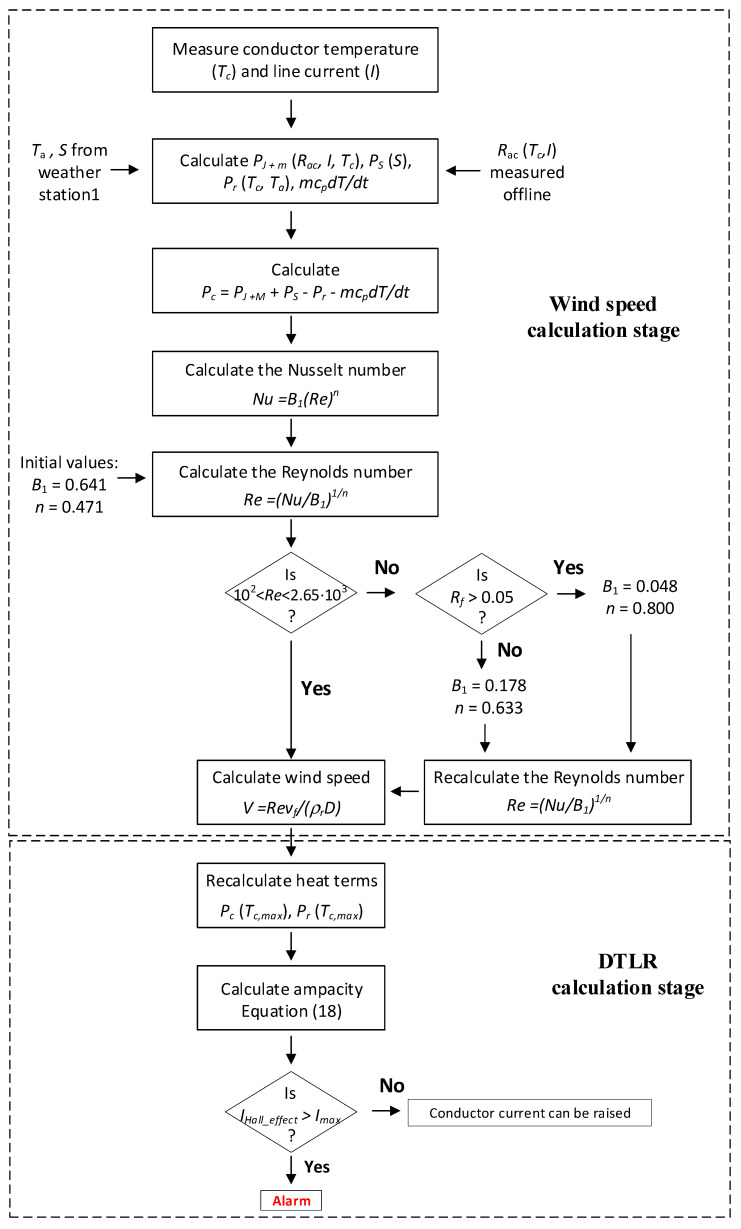
Proposed DTLR calculation method.

In case of forced convection, the Nusselt number is calculated as

(11) $$N_{u}=B_{1}{\left(Re\right)}^{n}\;\left[-\right]\;$$

where the Reynolds number is calculated as

(12) $$Re=\rho_{r}V\frac{D}{\nu_{f}}\;\left[-\right]$$

where *V* (m/s) is the wind speed,$\;\rho_{r}$ (−) and $\nu_{f}$ (m^2^/s) are the relative density and kinematic viscosity of air, respectively, *D* (m) is the diameter of the conductor, and *B*_1_ and *n* are constants depending on the Reynolds number *Re* and conductor surface roughness, respectively. It is worth noting that the wind speed can be estimated by applying (12), as detailed in Figure 2.

Table 1 provides the values of the coefficients *n* and *B*_1_, which depend on the Reynolds number and the surface roughness defined as *R_f_* = *d*/[2(*D* − *d*)], where *d* (m) is the diameter of the strands.

In case of natural cooling, the Nusselt number is obtained from the Grashof (*Gr*) and Prandtl (*Pr*) numbers as follows:

*Nu* = *A*_2_(*GrPr*)^m^_2_ (13)

*Pr* = 0.715 − 2.5 × 10^−4^*T_f_* (14)

*Gr* = *D*^3^(*T_c_ − T_a_*)*g*/(*T_f_* + 273)*v_f_*^2^ (15)

where *g* = 9.807 m/s^2^ and the values of *A*_2_ and *m*_2_ are found in Table 2.

The procedure described in this paper estimates the wind speed value. Thus, since the wind speed is not known, both forced and natural cooling equations are applied. If the power loss due to forced cooling is greater than the power loss due to natural cooling, it is assumed that the wind speed is not zero, and the Nusselt number *Nu* is calculated by applying (11); otherwise, it is calculated from (13). However, in virtually all situations found in outdoor environments, the Nusselt number must be calculated from (11).

The heat gain due to the solar radiation can be calculated using the global solar radiation *S* (W/m^2^), as seen in [7]:

(16) $$P_{s}=\alpha_{s}SD\;\left[W/m\right]$$

where *α*_s_ (-) is the solar absorptivity of the conductor surface whose value is assumed to be 0.5 [37], and *D* (m) is the external diameter of the conductor.

Finally, radiation heat losses can be described as [7]:

(17) $$P_{r}=\pi \varepsilon D\sigma_{B}\left[{\left(T_{c}+273\right)}^{4}-{\left(T_{a}+273\right)}^{4}]\;[W/m\right]\;$$

where ε is the emissivity factor, which depends on the conductor surface, and it is assumed to be 0.5 [4,37], and where *σ_B_* = 5.6697 × 10^−8^ W/(m^2^K^4^) is the Stefan–Boltzmann constant.

Finally, the DTLR rating is determined when the conductor temperature reaches it maximum value under thermal equilibrium, so from (6) and (8), it results in [5,28]:

(18) $$I_{max}=\sqrt{\frac{P_{c}\left(T_{c,max}\right)+P_{r}\left(T_{c,max}\right)-P_{s}}{R_{ac}\left(T_{c,max}\right)}}$$

## 4. Proposed Real-Time Method to Determine the Thermal Line Rating

The dynamic thermal line rating can not only be calculated by obtaining real-time weather data and load, but it can also be estimated several ways [26]. In this paper, a cost-effective, real-time monitoring model to calculate the DTLR rating using the *Smartconductor* is presented, the steps of which are described in Figure 2. This procedure has two main stages, i.e., the wind speed calculation stage and the DTLR calculation stage. The calculations associated with both stages are performed by the gateway. As constants *B*_1_ and *n* depend on surface roughness and Reynolds number, which are not available, this paper proposes setting their values to *B*_1_ = 0.641 and *n* = 0.471 in the initial stage, which are taken from [7] and are summarized in Table 1. In the first stage, the Reynolds number is corrected in order to estimate the wind speed. Next, the wind speed can be estimated, and if the maximum allowable conductor temperature is known (90 °C in this paper), then the ampacity can be predicted.

Finally, the predicted value of the ampacity ($I_{max}$) provided by (18) is compared to the measured current by the Hall effect sensor ($I_{HallSensor}$). In the case where *I_max_ < I_HallSensor_*, the current flowing through the line can be increased. Conversely, an alarm signal will be activated if $I_{max}>I_{HallSensor}$.

## 5. Experimental Setup

This section develops the experimental part of this paper to evaluate the accuracy and performance of the proposed approach for predicting the thermal line rating of power transmission lines.

The tests were performed in a high-current laboratory (AMBER laboratory from the Universitat Politècnica de Catalunya).

The analyzed ACSR conductor (550-AL1/71-ST1A, HAASE Gesellschaft mbh, Graaz, Austria) was supported by wood trestles and was connected to the output of the high-current transformer, forming a low-impedance loop.

**Figure 3:**
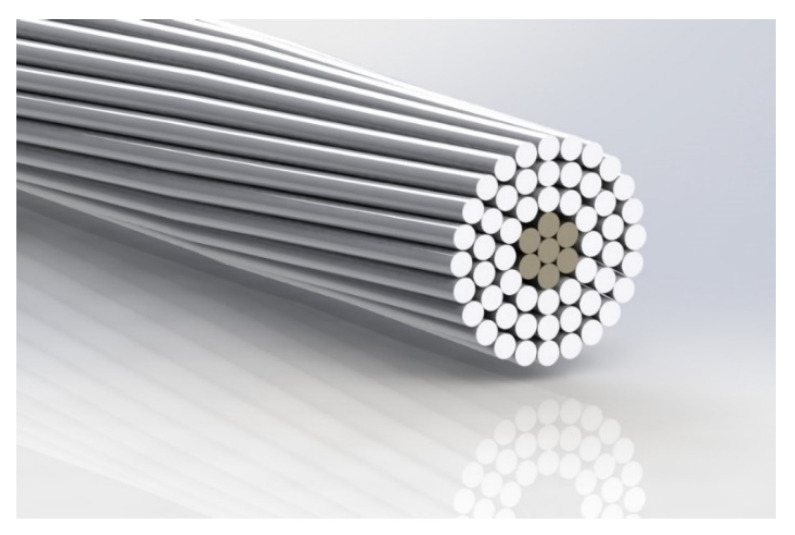
Cross section of the tested conductor.

Figure 3 shows the geometry of the 550-AL1/71-ST1A ACSR conductor that was used, including the 7 steel strands and the 54 aluminum strands, whereas Table 3 shows its main properties. 

Figure 3 details the geometry of the 550-AL1/71-ST1A ACSR conductor.

As explained in Section 3, in order to determine the heat gain due to the joule and magnetic heating, it is necessary to determine the evolution of the *R_ac_* resistance as a function of the conductor temperature. To this end, an experiment was performed off-line by measuring the voltage drop, temperature, cos*φ,* and current through 1 m of the analyzed conductor (550-AL1/71-ST1A ACSR conductor). The results that were obtained are summarized in Table 4. These values are required to evaluate (8).

Since the experiment was conducted indoors, two variable speed fans (V-6020 ROVEX, 50 W, 65 m^3^/min) and two dimmable linear led lamps (36 inch, 234 W, AUXTINGS, Foshan, China) were used to simulate the effect of wind and solar radiation, respectively.

The current and temperature of the cable were measured by the *Smartconductor* by means of the Hall effect sensor and the Pt1000 sensor, as described in Section 2.1 and Section 2.2, respectively.

Regarding the Hall effect sensor, considering several parameters such the possibility of being integrated with microelectronics, performance efficiency, accuracy, cost, and size, the DRA5053 analog-bipolar Hall effect sensor from Texas Instruments (Dallas, TX, USA) [38] was selected for this application [39,40,41].

Regarding the Pt1000 sensor, the PTFC102T1G0 sensor from TE connectivity (Schaffhausen, Switzerland) is a suitable choice [42] because it has a rated resistance of 1000 Ω to provide typical accuracies of ±0.1 °C with a temperature range between −30 °C to 200 °C.

To measure the wind speed, an anemometer (RH Anemometer Pen 850021, Sper Scientific, Scottsdale, AZ, USA) with a measuring range of 0.4–30 m/s with a resolution of 0.1m/s and an accuracy of 3% full scale when the wind speed is below 20 m/s was used.

Finally, to measure the solar radiation, a solar power meter (PCE-SPM1, Professional Calibrated Equipments, PCE, Tobarra, Spain) was used. It had a measuring range between 0–2000 W/m^2^, a resolution of 0.1 W/m^2^, and an accuracy of ±10 W/m^2^.

To validate and check the accuracy of the results provided by the *Smartconductor*, the temperature and the current of the conductor were measured using a T-type thermocouple connected to a thermocouple input module (NI-9211, National Instruments, Dallas, TX, USA) and a Rogowski coil (500LFxB from PEM, Nottingham, UK with sensitivity 0.06mV/A) connected to a data acquisition system (NI USB-6356 DAQ, National Instruments, Dallas, TX, USA, with eight differential inputs). For simultaneous acquisition, the NI-9211 thermocouple input module and the NI USB-6356 DAQ were synchronized by means of a Python code. The data from the two DAQs were synchronized with the data from the *Smartconductor* by means of a MATLAB^®^ code.

**Figure 4:**
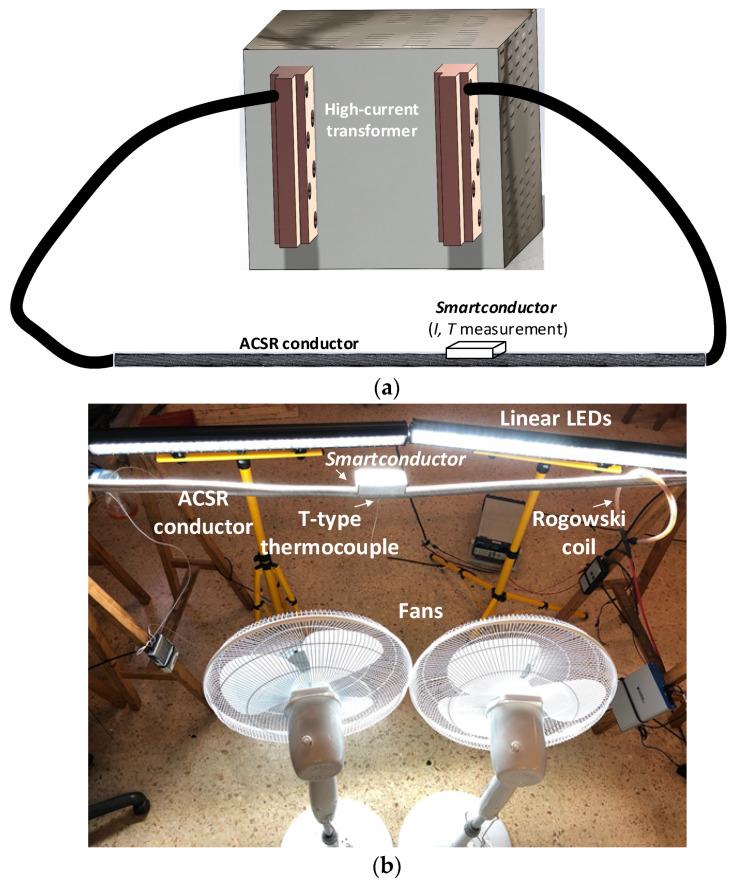
(**a**) Sketch of the experimental setup; (**b**) detail of the ACSR conductor and the sensors.

Figure 4 shows the experimental setup, including the conductor loop, the high-current transformer, and the sensors used to validate the method proposed in this paper to determine the wind speed and the DTLR rating.

## 6. Experimental Results

### 6.1. First Experiment. Wind Speed and DTLR Estimation

**Figure 5:**
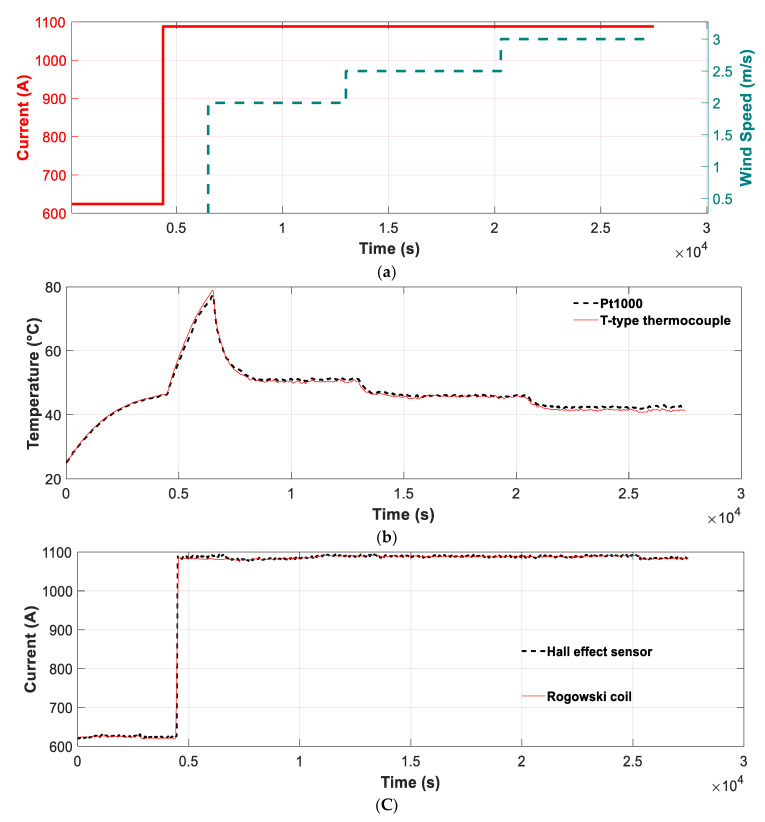
(**a**) Planned current intensity and perpendicular wind speed profiles during the test. (**b**) Conductor temperature measured by the Pt1000 sensor (*Smartconductor*) and the T-type thermocouple. (**c**) Currents measured by the Hall effect sensor (*Smartconductor*) and the Rogowski coil.

A first experiment that was conducted to determine the accuracy of the proposed method in estimating the wind speed and DTLR of the studied conductor is shown in Figure 5. To this end, a current change (from around 600 A to around 1100 A) was applied to the loop shown in Figure 4, and four wind speeds were applied (0 m/s, 2 m/s, 2.5 m/s and 3 m/s) as shown in Figure 5a. During these tests, the solar radiation was set to a constant value of 800 W/m^2^.

Figure 5b shows the temperature measured by the PTC1000 incorporated in the *Smartconductor* and by the laboratory sensor (T-type thermocouple), whereas Figure 5c shows the current measured by the Hall effect sensor and the Rogowki coil under the conditions established in Figure 5a. These results show that the temperature and current measurements made with the *Smartconnector* sensors and the laboratory measurements are very similar, thus validating the accuracy of the *Smartconductor* measurements.

The average difference of the temperature measured by the Pt1000 sensor included in the *Smartconductor* compared to the measurement of the laboratory device (T-type thermocouple) is 1.34%, whereas the maximum difference is 3.48%. The average difference of the current measured by the Hall effect sensor compared to the measurements of the laboratory device (Rogowski coil) is 0.23%, whereas the maximum difference is 1.92%; thus, the *Smartconductor* shows reliable and accurate results.

**Figure 6:**
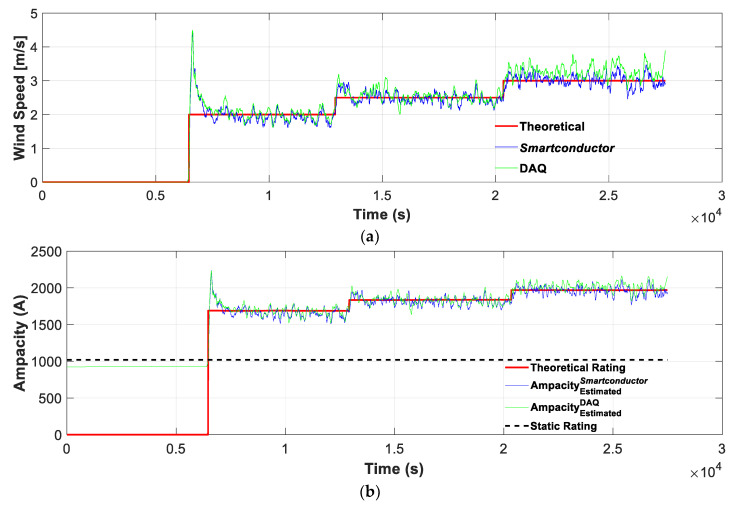
(**a**) Theoretical and estimated wind speeds with the *Smartconductor* (using the Pt1000 and Hall effect sensor) and the DAQ (using T-type thermocouple and Rogowski coil); (**b**) static and theoretical and estimated ampacity values with the *Smartconductor* (using the Pt1000 and Hall effect sensor) and the DAQ (using T-type thermocouple and Rogowski coil).

Figure 6 compares the wind speed and the DTLR estimates provided by the *Smartconductor* and the laboratory measurements with the theoretical values. These estimates are based on the conditions shown in Figure 5. The results presented in Figure 6 show very similar results, thus validating the proposed methodology.

It is worth noting that the theoretical rating *I_max_* (red line in Figure 6b) was obtained from (18) by taking into account the measured values (real values) of the wind speed.

Table 5 summarizes the results that were attained. It shows that the estimated wind speeds are very close to the applied ones and that the estimated ampacities at the different wind speeds are very close to the theoretical values, which were calculated by applying (18) and considering the measured values of the wind speed instead of the ones that were estimated by the method proposed in this work since the differences are below 2.3%.

### 6.2. Second Experiment. Validation of the Accuracy of the Proposed Method to Estimate the DTLR

**Figure 7:**
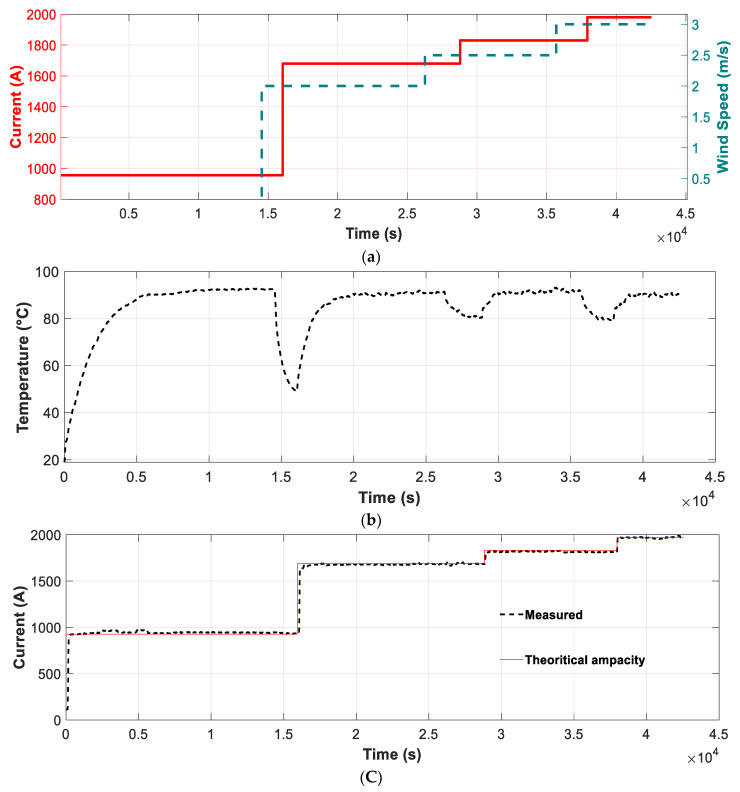
(**a**) Current and wind speed profiles during the test. (**b**) Conductor temperature measured by the T-type thermocouple and the Pt1000 sensor; (**c**) Applied and theoretical current values.

A second experiment was conducted to validate the accuracy of the DTLR estimation method proposed in this paper. To this end, the current and wind speed profiles shown in Figure 7a were applied to the analyzed conductor. The values of the applied currents were selected so that the equilibrium conductor temperature was 90 °C under the four wind conditions (0 m/s, 2 m/s, 2.5 m/s and 3 m/s), i.e., the maximum allowable temperature of the tested ACSR conductor for continuous operation. Thus, the same laboratory setup as the one used in the previous tests was used, and four current levels were injected (956 A, 1680 A, 1830 A, and 1980 A, which correspond to the four wind speeds 0 m/s, 2 m/s, 2.5 m/s and 3 m/s, respectively) to heat the ACSR conductor up to 90 °C. The results that were attained are shown in Figure 7.

Table 6 summarizes the numerical values corresponding to Figure 7. These results show that the difference between the real and estimated currents needed to bring the conductor to the maximum allowable temperature is low and are always below 3.0%, thus validating the method proposed in this paper.

Regarding the computational requirements of the proposed approach, the estimation of the wind speed requires 0.05 ms and the estimation of the DTLR requires 0.07 ms when using a Intel(R) Xeon(R) CPU E5-2620 v4 processor with 64 Gb RAM memory

## 7. Conclusions

This paper has presented a real-time monitoring system to determine the ampacity of ACSR conductors, called a *Smartconductor*. To this end, the actual values of the ambient temperature and solar radiation are required and can be obtained from a nearby weather station, whereas the *Smartconductor* measures the current and temperature of the conductor. Since the wind speed at the conductor surface greatly depends on its exact location and since it has an important cooling effect, it is essential to have an accurate estimation of the local wind speed at the conductor. Therefore, a method that accurately estimates the wind speed has also been presented. Once this parameter is known, the approach presented in this paper allows the calculation of the DTLR rating of the analyzed conductor based on a thermal model. To validate the accuracy and performance of the approach presented in this paper, different situations have been tested in the laboratory using ACSR conductors by controlling and measuring the solar radiation, wind speed, local temperature, conductor temperature, and line current. The proposed approach also includes a method to estimate the combined joule and magnetic losses of the ACSR conductor from the ac resistance.

The experimental results presented in this paper prove that the real-time approach presented in this paper can predict both the value of the local wind speed and the DTLR with accuracy while requiring a reduced computational burden, so the calculations can be implemented in low-power microprocessors used in inexpensive devices that are required for a global deployment of smart grids. Therefore, the findings in this paper contribute to research concerning smart grids. The proposed DTLR approach allows us to take advantage of the maximum transmission capacity of power lines by adapting the rating of the line according to the current weather conditions in real-time, making it a smart solution that is of paramount importance for future smart grids.

## Figures and Tables

**Table 1:** Values of constants *n* and *B*_1_ [7].

Surface Type	*Re*	*n*	*B*_1_
All surfaces stranded	100–2650	0.471	0.641
Stranded *R_f_* ≤ 0.05	2650–50,000	0.633	0.178
Stranded *R_f_* ≥ 0.05	2650–50,000	0.800	0.048

**Table 2:** Values of the product *Gr·Pr* [7].

*Gr·Pr*	*A*_2_	*m*_2_
10^2^–10^4^	0.850	0.188
10^4^–10^6^	0.480	0.250

**Table 3:** Parameters of the tested ACSR conductor 550-AL1/71-ST1A.

Symbol	Description	Value	Unit
$A_{Al}$	Area of aluminum	549.7	${\mathrm{mm}}^{2}$
$A_{steel}$	Area of steel	71.3	${\mathrm{mm}}^{2}$
$N_{Al}$	Number of aluminum wires	54	-
$N_{Steel}$	Number of steel wires	7	-
$D_{Al},D_{steel}$	Aluminum and steel wire diameter	3.6	mm
D	Diameter of conductor	32.4	mm
$m_{AL}$	Mass per unit length of aluminum	1.5183	Kg/m
$m_{steel}$	Mass per unit length of steel	0.5583	Kg/m
$Cp_{aluminum}$	Specific heat of aluminum	897	J/(Kg°C)
$Cp_{steel}$	Specific heat of steel	481	J/(Kg°C)
$R_{20{}^{\circ}C}$	DC resistance of the conductor	0.0526	Ω/km
$I_{max}$	Current carrying capacity	1020	A

**Table 4:** Dependence of *R_ac_* with the temperature of the conductor.

T (°C)	Voltage Drop (V_RMS_)	Current (A_RMS_)	cos*φ*	*R_ac_* (µΩ/m)
30	0.10	1025	0.59	57.7
40	0.10	1022	0.60	60.2
50	0.10	1023	0.62	62.4
60	0.10	1022	0.63	64.8
70	0.10	1026	0.64	67.2
80	0.11	1028	0.65	69.4
90	0.11	1023	0.67	71.6
100	0.14	1305	0.68	74.3

The *R_dc_* resistance at 30 °C is 56.7 µΩ/m.

**Table 5:** Results of estimated wind speed and ampacity predicted by the proposed approach.

Current (A) (%Static Rating)	Theoretical Wind Speed (m/s)	Theoretical Line Rating (A)	Average Estimated Wind Speed (m/s)by *Smartconductor*	Average Estimated Wind Speed (m/s)by DAQ System	Average Estimated Ampacity(A) by *Smartconductor*	Average Estimated Ampacity (A) by DAQ	Error of Line Rating Calculation by *Smartconductor* (%)	Error of Line Rating Calculation by DAQ System (%)
624 (55%)	0	927	0	0	927	927	0.0	0.0
1088 (97%)	2	1688	1.90	1.99	1648	1670	2.3	1.0
1088 (97%)	2.5	1833	2.48	2.53	1813	1830	1.0	0.2
1088 (97%)	3	1969	3.03	3.28	1961	2016	0.2	2.3

The static rating of the conductor is 1020A.

**Table 6:** Results of steady-state temperature with different currents and wind speeds.

Currents			Wind Speed (m/s)	Steady-State Conductor Temperature (°C)
Applied (A)	Estimated (A)	Difference (%)		
956	927	3.0	0.0	Around 90
1680	1688	0.5	2.0	Around 89
1830	1833	0.2	2.5	Around 90
1980	1969	0.6	3.0	Around 89
